# The scholarly footprint of ChatGPT: a bibliometric analysis of the early outbreak phase

**DOI:** 10.3389/frai.2023.1270749

**Published:** 2024-01-05

**Authors:** Faiza Farhat, Emmanuel Sirimal Silva, Hossein Hassani, Dag Øivind Madsen, Shahab Saquib Sohail, Yassine Himeur, M. Afshar Alam, Aasim Zafar

**Affiliations:** ^1^Department of Zoology, Aligarh Muslim University, Aligarh, India; ^2^Department of Economics and Law, Glasgow School for Business and Society, Glasgow Caledonian University, Glasgow, United Kingdom; ^3^The Research Institute of Energy Management and Planning (RIEMP), University of Tehran, Tehran, Iran; ^4^USN School of Business, University of South-Eastern Norway, Hønefoss, Norway; ^5^Department of Computer Science and Engineering, School of Engineering Sciences and Technology, Jamia Hamdard, New Delhi, India; ^6^College of Engineering and Information Technology, University of Dubai, Dubai, United Arab Emirates; ^7^Department of Computer Science, Aligarh Muslim University, Aligarh, India

**Keywords:** ChatGPT, bibliometric analysis, scientometric methods, research trends, citation analysis, collaborative networks, application domains, future directions

## Abstract

This paper presents a comprehensive analysis of the scholarly footprint of ChatGPT, an AI language model, using bibliometric and scientometric methods. The study zooms in on the early outbreak phase from when ChatGPT was launched in November 2022 to early June 2023. It aims to understand the evolution of research output, citation patterns, collaborative networks, application domains, and future research directions related to ChatGPT. By retrieving data from the Scopus database, 533 relevant articles were identified for analysis. The findings reveal the prominent publication venues, influential authors, and countries contributing to ChatGPT research. Collaborative networks among researchers and institutions are visualized, highlighting patterns of co-authorship. The application domains of ChatGPT, such as customer support and content generation, are examined. Moreover, the study identifies emerging keywords and potential research areas for future exploration. The methodology employed includes data extraction, bibliometric analysis using various indicators, and visualization techniques such as Sankey diagrams. The analysis provides valuable insights into ChatGPT's early footprint in academia and offers researchers guidance for further advancements. This study stimulates discussions, collaborations, and innovations to enhance ChatGPT's capabilities and impact across domains.

## 1 Introduction

The rapid advancements in artificial intelligence (AI) have led to the development of sophisticated language models that can understand and generate human-like text. One such notable AI language model is ChatGPT (https://openai.com/chatgpt), an autoregressive language model that uses deep learning techniques to generate coherent and contextually relevant responses to user inputs. Since its launch, ChatGPT has gained significant attention and adoption in various domains, includinrg content generation, healthcare, education, data science, accounting, finance, tourism, and customer support/assistance (Carvalho and Ivanov, 2023; Dowling and Lucey, 2023; Dwivedi et al., 2023a,b; Gupta et al., 2023; Ray, 2023; Sallam, 2023; Sohail et al., 2023a; Wood et al., 2023). The introduction of ChatGPT has also sparked discussions and debates surrounding its potential implications across various domains (Baumgartner, 2023; Ivanov and Soliman, 2023; Lo, 2023). Notably, issues related to ethical considerations and biases (Ray, 2023; Sohail et al., 2023b) and the impact of large language models on knowledge assessment (Farhat et al., 2023b; Gilson et al., 2023) have garnered attention in recent discourse. Scholarly investigation of ChatGPT has emerged as a critical area of research, aiming to understand its impact, applications, and future directions (Dave et al., 2023; Gupta et al., 2023; Ray, 2023; Roumeliotis and Tselikas, 2023; Sohail et al., 2023a).

To date, however, only a few authors have used bibliometric and scientometric methods to analyze ChatGPT. Khosravi et al. (2023) carried out an analysis of the broader chatbot literature, while Levin et al. (2023) used bibliometrics to explore publications on ChatGPT in the field of obstetrics and gynecology. Our study differs by focusing especially on ChatGPT publications and by considering the latest developments up until June 2023. In our view, bibliometric and scientometric analysis can provide valuable insights into the research landscape surrounding ChatGPT, including the evolution of research outputs, citation patterns, collaborative networks, application domains, and emerging research trends. By analyzing a comprehensive dataset of scholarly publications, this study aims to shed light on the scholarly footprint of ChatGPT and its influence in academia.

This study employs a multifaceted approach, utilizing bibliometric and scientometric methods to analyze the scholarly footprint of ChatGPT. Bibliometric analysis (see, for example Donthu et al., 2021) offers a quantitative approach to evaluate the scholarly impact of ChatGPT research. By carefully gathering and analyzing relevant data from the Scopus database, we address several pivotal research questions:

➢ Publication trends: How has research output related to ChatGPT evolved over time? What are the prominent publication venues and journals that feature research on ChatGPT?
➢ Citation analysis: How has ChatGPT been referenced in scholarly literature? Which papers, authors, countries, and journals have made significant contributions to the understanding and advancement of ChatGPT?
➢ Collaborative networks: Who are the key contributors and collaborators in the ChatGPT research landscape? What patterns of collaboration and co-authorship exist among researchers, institutions, and countries working on ChatGPT-related topics?
➢ Application domains: In which primary domains has ChatGPT found an application? How are researchers leveraging its capabilities in fields such as customer support, content generation, and virtual assistance?
➢ Future directions: Based on the keyword analysis related to ChatGPT's scholarly footprint, what emerging keywords and potential research areas for future research can be identified? What challenges and opportunities lie ahead in enhancing ChatGPT's capabilities and impact?

By addressing these research questions, this paper aims to provide a comprehensive and up-to-date analysis of ChatGPT's scholarly footprint. Our findings not only contribute to a better understanding of ChatGPT's influence in academia but also serve as valuable insights for researchers interested in the development and utilization of AI language models. Ultimately, through mapping its progress and identifying future trends, we aim to stimulate discussions, collaborations, and innovations that drive the continued advancement of ChatGPT and its applications across various domains.

By utilizing data from the Scopus database, we identified 533 relevant articles published between November 2022 and early June 2023 that focus on ChatGPT. The selected articles underwent thorough evaluation based on various criteria, including organization, country/region, journal, total citations, and keywords. This analysis revealed several key insights, as presented in our findings and discussion later on. For example, there has been a remarkable surge in scholarly publications related to ChatGPT, with 533 articles produced within a short span of 6 months. This indicates a thriving research interest and highlights the growing recognition of the potential applications of ChatGPT. Furthermore, the high collaboration rate of 88.91% among authors suggests a strong community of researchers working on ChatGPT, sharing ideas and resources to advance the field. In addition, we also uncover interesting details about the publication venues contributing to ChatGPT research, which evidences its impact in diverse scientific disciplines, the contributions of different countries to ChatGPT research, and top authors and institutions.

The remainder of this paper is organized such that Section 2 presents the methodology, and Section 3 provides an overview of the main findings. Section 4 discusses the findings in relation to the existing literature on ChatGPT. Finally, Section 5 concludes the paper by highlighting contributions, limitations, and directions for further research.

## 2 Methodology

### 2.1 Data extraction

**Figure 1:**
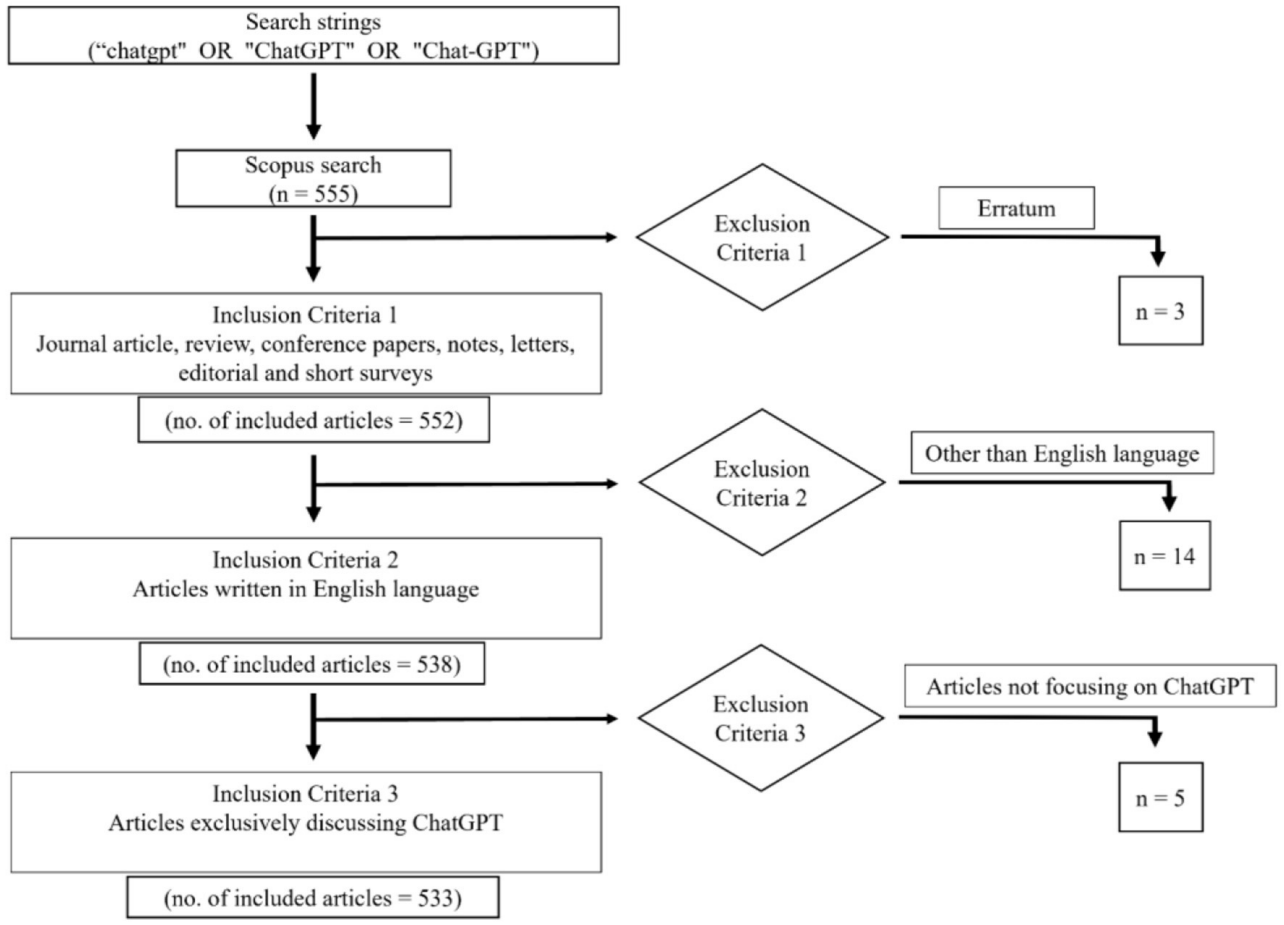
Methodology flow chart.

Figure 1 provides an overview of the methodology. On June 6, 2023, we used the Scopus database to search for articles that contain the search queries “chatgpt,” “ChatGPT,” or “Chat-GPT” in the title, abstract, or keywords. By employing the specified keywords, 555 articles were initially retrieved. Following the initial exclusion criteria, which involved omitting “errata,” the remaining count was reduced to 552 articles encompassing various types such as journal articles, reviews, notes, conference papers, letters, editorials, and short reviews. Subsequently, we applied the second exclusion criterion, which involved excluding articles in languages other than English, resulting in a final selection of 538 articles written in English. These chosen papers were then subjected to a meticulous analysis, during which the third exclusion criterion was employed to remove articles that did not exclusively focus on the topic of ChatGPT. The selected 533 articles are used for our bibliometric review study and evaluated using the following criteria: organization, country/region, journal, total citations, and keywords. We downloaded the complete records for bibliometric analysis and imported them into the Biblioshiny (Bibiliometrix) and VOSviewer software packages. Biblioshiny was employed for the overview analysis of the retrieved documents, relevant keyword analysis, and plotting the three-field Sankey Diagrams. VOSviewer software was used to illustrate various collaboration networks, perform citation and co-citation analysis, and identify the top five keywords along with their co-occurrence networks. Various indicators have been used in the literature for bibliometric analysis, including total article count, average citations per article (ACPA), total citation count, total link strength, and Hirsch index (H-index). These metrics are commonly used in bibliometric studies, with the H-index being a widely recognized measure of research quality and quantity for authors and research areas (Farhat et al., 2023a). ACPA is also widely accepted as a measure of research impact for individual works, authors, and publication outlets. Citation analysis is conducted to explore the scientific impact and themes of the study under consideration, and co-authorship and co-occurrence have also been investigated to analyze scientific collaboration. Three-field Sankey diagrams are also used to identify the relationship among three interrelated sets of values (Aria and Cuccurullo, 2017). All of these indicators have been taken into account in this bibliometric study.

### 2.2 Data analysis

In conducting our bibliometric analysis of ChatGPT research, several critical assumptions underpin our interpretations. Firstly, we have considered Scopus as a foundational dataset for our work, and we assume the completeness and representativeness of our dataset, trusting that it adequately captures the vast landscape of publications in the field. Simultaneously, we acknowledge the inherent challenge of achieving absolute comprehensiveness and recognizing potential omissions or biases. Excluding other datasets, for example, Google Scholar, may cause the omission of a few articles. However, on the other hand, Scopus provides unbiased, reliable, and standard articles from reputed journals. Our analysis relies on the accuracy of metadata, presuming that author names, publication titles, and affiliations are error-free. Furthermore, we assume consistency in citation practices across publications and authors, understanding that variations can exist and impact citation-based analyses. The representativeness of citation metrics, temporal patterns, and research topics within the dataset is also assumed, acknowledging the potential for variations and heterogeneity. We also assume that the dataset is current, reflecting the contemporary state of the field. Finally, we operate under the assumption that the data used in our analysis adheres to ethical standards, with due consideration for potential biases or ethical concerns related to our data sources.

In light of these assumptions, a comprehensive analysis of ChatGPT research was conducted, encompassing 533 publications from 87 countries and 1,195 institutions. These publications, originating from 341 different sources, were authored by 1,434 individuals and received a total of 1,362 citations. Moreover, a total of 1,998 keywords were identified. The analysis involved employing the full counting approach, which focuses on elements connected to one another. This approach facilitated citation analysis and co-authorship analysis. Collaboration networks among authors, institutions, and countries were visualized using illustration maps. The size of the circles in these maps indicates the strength and frequency of collaborations between individuals and organizations. The connecting lines among these circles, termed “links,” represent the connections or relationships between various elements, such as authors, documents, or keywords. These links can represent co-authorships, co-citations, or co-occurrences of keywords in a bibliometric network. “Total Link Strength” is a metric used in network analysis and refers to the overall strength or significance of connections within a network. It measures the combined influence or importance of all the links or connections between nodes or entities in a network.

Additionally, citation maps displayed the connections and citations between different partners, with larger circles representing higher citation counts and stronger linkages. To analyze the relationships between keywords, a keyword map was generated using the complete counting method. To examine the interactions among three distinct interconnected variables, three-field Sankey diagrams were utilized. These diagrams enable the analysis of relationships involving authors, author's keywords, and keywords. Similarly, the interplay between country, publication source, and keywords, as well as author, title-term, and source, were also investigated using these diagrams. Furthermore, the research trends and popular topics in ChatGPT research were explored through the identification of significant research terms, word cloud analysis, and examination of keyword co-occurrence. This map grouped related keywords together and assigned equal weight to each co-occurrence link. Consequently, terms with higher frequency were represented by larger circles in the map.

## 3 Findings

We begin by providing a comprehensive overview of the research conducted on ChatGPT during the period of 2022–2023. In a short span of only 6 months (November 2022 to early June 2023), a total of 533 documents were produced from 341 sources from 87 different countries involving 1,434 authors, indicating a thriving research interest (see Supplementary material). The total corpus involved 1,434 authors, with 159 of them contributing to single-authored documents. This represents a significant collaboration rate of 88.91%, highlighting the collaborative network within the research area. This collaboration suggests that there is a strong community of researchers working on ChatGPT, and that they are sharing ideas and resources to advance the field. Among the documents, 420 were single-country contributions, while 113 demonstrated collaboration between multiple countries. The involvement of 1,195 institutions highlighted diverse organizational contributions.

### 3.1 Types of documents published and the thematic area of research

**Figure 2:**
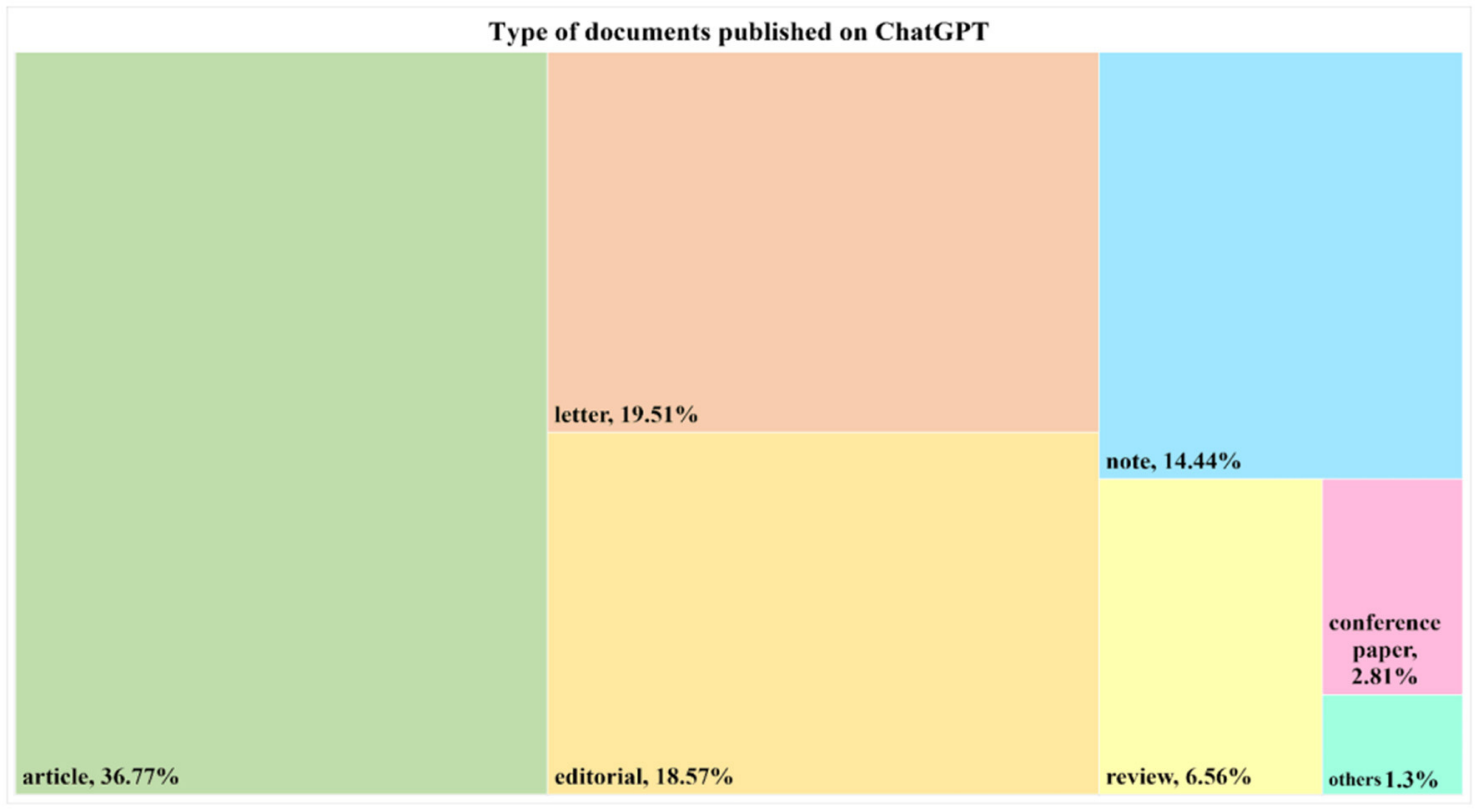
Tree map representing the type of documents published on ChatGPT.

**Figure 3:**
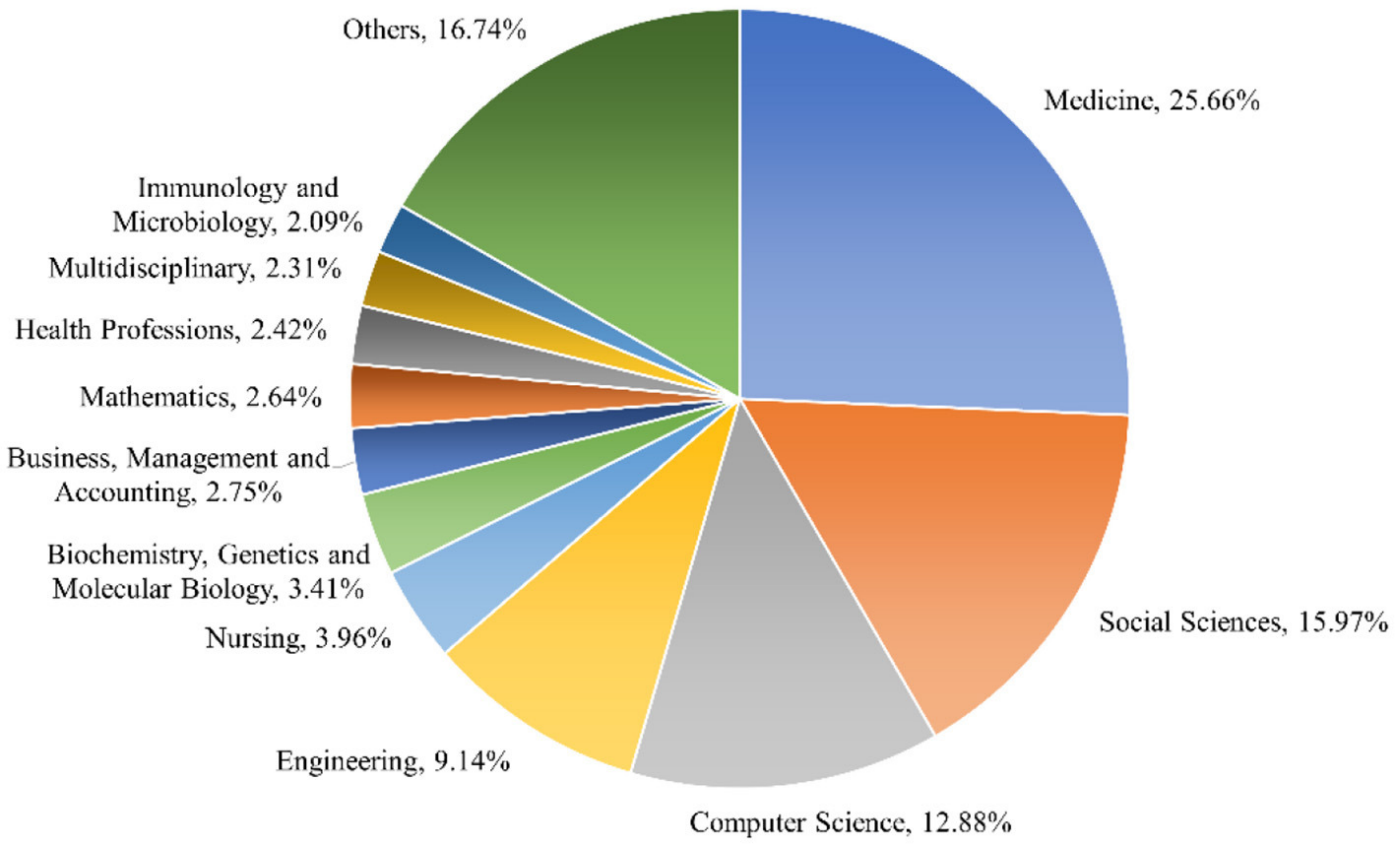
Thematic subject categories of research on ChatGPT.

This section provides an overview of the distribution of document types represented in ChatGPT research. Both traditional documents (e.g., articles, reviews, notes, and conference papers) and other documents, such as editorials and letters, are often not subject to peer review. Out of the total of 533 publications obtained, a considerable portion comprises empirical papers, representing 36.77% (196 articles) of the corpus. Letters constitute 19.51% (104 articles), editorials make up 18.57% (99 publications), and notes account for 14.55% (Figure 2). Interestingly, it is observed that besides empirical papers, a substantial portion of the ChatGPT corpus consists of letters, notes, and editorials, making up 52.63% of the total publications. On the other hand, the number of review articles published was relatively low. Figure 3 provides an overview of the thematic subject categories of ChatGPT research. It can be seen that the most frequent categories are medicine, social sciences, computer science, and engineering.

There are 533 publications relating to ChatGPT in 341 different journals. The top 10 journals account for 17.63% of the corpus and 44.65% of the total citations. As Table 1 shows, Annals of Biomedical Engineering has thus far published the most articles (28), followed by Nature (17) and Library Hi Tech News (12). Nature was the most cited journal (367 citations), followed by Radiology (100) and Science (93). Based on their H-index, Nature ranks first (1,331), Radiology ranks second (320), and Annals of Surgical Oncology ranks third (192).

**Table 1:** Top 10 most relevant journals based on article count.

**Journal**	**Article count**	**Citation count**	**Average Citation per Article (ACPA)**	**H-index**	**Publisher**
Annals of Biomedical Engineering	28	46	1.64	150	Springer Netherlands
Nature	17	367	21.58	1,331	Nature Publishing Group
Library Hi Tech News	12	20	1.66	22	Emerald Group Publishing Ltd.
Medical Teacher	6	3	0.5	131	Informa Healthcare
Radiology	6	100	16.66	320	Radiological Society of North America Inc.
Accountability in Research	5	11	2.2	35	Taylor and Francis Ltd.
Annals of Surgical Oncology	5	1	0.2	192	Springer New York
IEEE/CAA Journal of Automatica Sinica	5	19	3.8	67	IEEE Advancing Technology for Humanity
JMIR Medical Education	5	39	7.8	23	JMIR Publications Inc.
Journal of Chemical Education	5	0	0	95	American Chemical Society

A total of 87 different countries have contributed to research on the topic of ChatGPT. Table 2 displays the top 10 countries on the basis of article count. The USA ranked first in terms of publications, with 173 articles, accounting for over 32.54% of the entire corpus. India (48 articles, 9%) and the UK (47 articles, 8.81%) rank second and third, respectively, in terms of contribution. In addition, the USA has a more considerable global academic impact than any other country, as demonstrated by the highest citation count (391). The UK ranks second in terms of citations with 153. Moreover, countries such as Australia, China, and Italy have made significant contributions, with citation counts of 76, 73, and 67, respectively.

**Table 2:** Top 10 most relevant countries based on article count.

**Country**	**Article count**	**Total citations**	**Average Citation per Article (ACPA)**
United States	173	391	2.26
India	48	50	1.04
United Kingdom	47	153	3.25
China	43	73	1.69
Australia	38	76	2
Canada	23	25	1.08
Italy	23	67	2.91
Germany	21	59	2.80
South Korea	15	33	2.2
France	14	57	4.07

Table 3 presents the top 10 authors and their corresponding article metrics. Wang F.Y. from the Institute of Automation Chinese Academy of Sciences in China stands out with the highest article count of 9 and a total of 23 citations. Following closely is Wu H. from Duke University School of Medicine in the USA, with seven articles and 16 citations. Interestingly, Kleebayoon A. from Joseph Ayo Babalola University in Nigeria and Wiwanitkit V. from Chandigarh University in India have published 6 articles each but have not received any citations. On the other hand, authors with a lower article count have also garnered significant citation numbers. For instance, Ali M. J. from L. V. Prasad Eye Institute in India, Lu Y. from Zhengzhou University in China, and Gu S. from Duke University School of Medicine in the USA have achieved 8, 10, and 15 citations, respectively, with just five articles each. Among the top 10 authors, three authors represent China and three represent the USA, while two hail from India.

**Table 3:** Top 10 most relevant authors based on article count.

**Authors**	**Article count**	**Total citations**	**Average Citation per Article**	**Affiliation**	**Country of origin**
Wang F.Y.	9	23	2.55	Institute of Automation Chinese Academy of Sciences	China
Wu H.	7	16	2.28	Duke University School of Medicine	USA
Cheng K.	6	12	2	Zhengzhou University	China
He Y.	6	16	2.66	The University of North Carolina at Chapel Hill	USA
Kleebayoon A.	6	0	0	Joesph Ayobabalola University	Nigeria
Teixeira Da Silva J.A.	6	5	0.83	Miki-cho Post Office, Kagawa	Japan
Wiwanitkit V.	6	0	0	Chandigarh University	India
Ali M.J.	5	8	1.6	L.V. Prasad Eye Institute India	India
Gu S.	5	15	3	Duke University School of Medicine	USA
Lu Y.	5	10	2	Zhengzhou University	China

A total of 1,195 institutions have contributed to the 533 publications, with Duke University participating in the most papers (14). Chinese Academy of Sciences (9) and Chandigarh University (8) make up the top three organizations based on article count (Table 4). Duke University has received the most citations, cited 32 times, followed by Chinese Academy of Sciences and the University of Chinese Academy of Sciences with 23 citations each. In terms of average citations per article, the University of Chinese Academy of Sciences takes the top position with 3.28, followed by the Beijing Sport University with 2.66. Among the top 10 institutions, five institutions are from China, representing the highest contribution to the field, and two are from the USA.

**Table 4:** Top 10 most relevant Institutions based on article count.

**Organization**	**Article count**	**Total citations**	**Average Citation per Article**	**Country of origin**
Duke University	14	32	2.28	USA
Chinese Academy of Sciences	9	23	2.55	China
Chandigarh University	8	02	0.25	India
Johns Hopkins School of Medicine	7	08	1.14	USA
Tianjin Medical University	7	16	2.28	China
University of Chinese Academy of Sciences	7	23	3.28	China
Beijing Sport University	6	16	2.66	China
University of Toronto	6	03	0.5	Canada
Zhengzhou University	6	12	2	China
Monash University	6	06	1	Australia

### 3.2 Most cited documents, authors, countries, and journals

**Figure 4:**
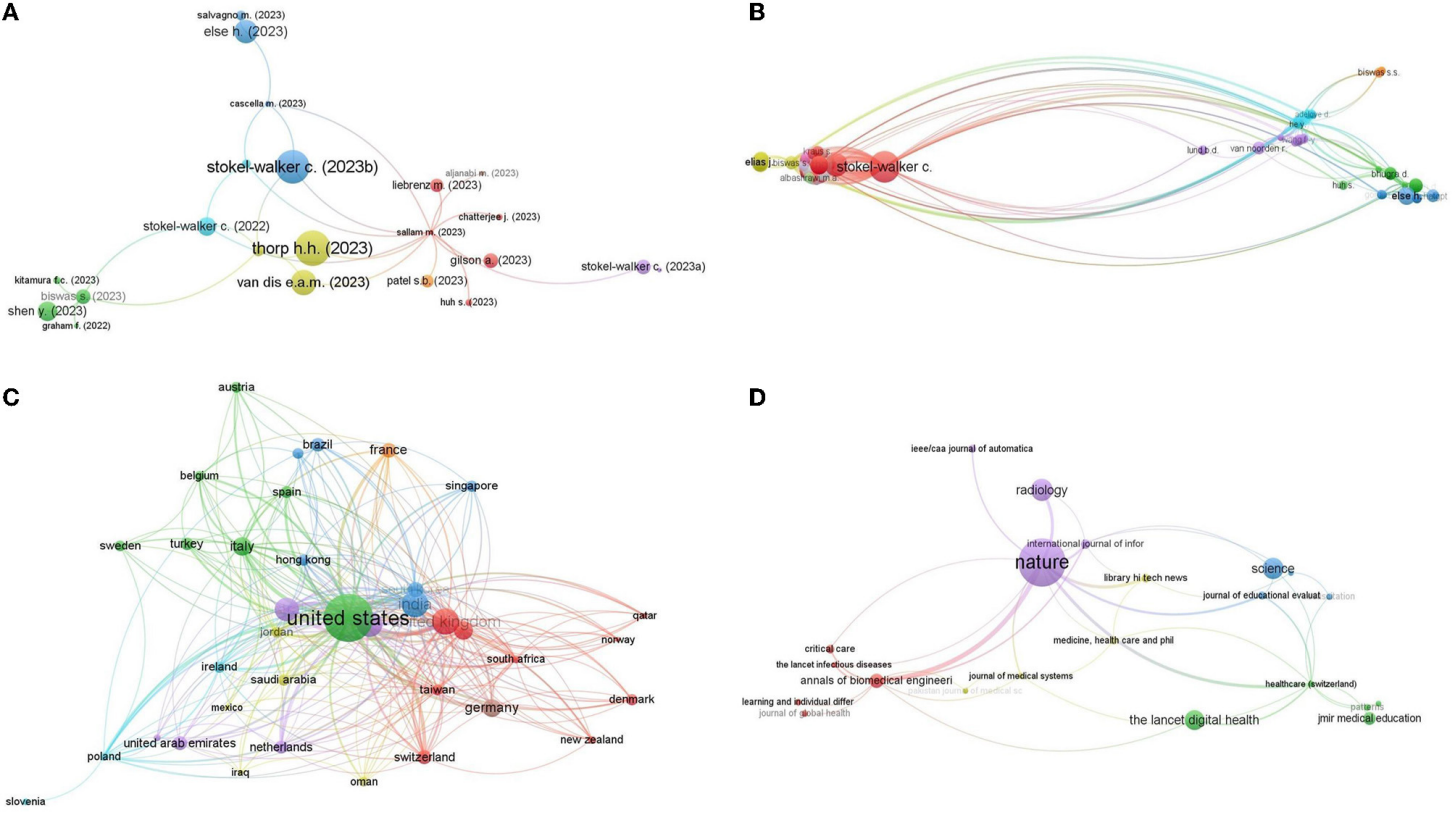
**(A)** Most cited articles and their citation network. **(B)** Most cited authors and their citation network. **(C)** Most cited countries and their citation network. **(D)** Most cited journals and their citation network.

When the citation network analysis was carried out in VOSviewer, it was observed that 34 articles have at least 10 citations, 15 articles have 20 citations, and only five articles have received 50 citations (Figure 4A). The size of the circle denotes the number of citations, and the connecting lines represent their citation network. The larger the circle larger the citation count of an article, and the more connecting lines reflect that the articles are citing another article or cited by other articles (Van Eck and Waltman, 2010). A total of 22 articles organized in 8 different clusters are linked among each other with 28 links (Figure 4B). The largest citation network is associated with Sallam (2023) with 13 links, followed by Biswas (2023) and Dwivedi et al. (2023a) with five links independently. The most cited document in the field of ChatGPT research is the editorial titled “ChatGPT is fun, but not an author” by Thorp (2023), with 93 citations. The second most cited document is a note titled “ChatGPT listed as an author on research papers: many scientists disapprove” by Stokel-Walker (2023), with 88 citations. These two influential works have significantly raised awareness and initiated critical conversations about the ethical implications of attributing authorship to AI language models.

The top 20 most cited documents are listed in Table 5. Of the top 20 most cited documents, eight are notes, six are editorials, four are articles, and only two are reviews. The only research article that is among the top 10 most cited documents is “How Does ChatGPT Perform on the United States Medical Licensing Examination? The Implications of Large Language Models for Medical Education and Knowledge Assessment” (Gilson et al., 2023), otherwise it is either a note or editorial that forms the top 10 most cited document list. Seven of the top 20 most cited documents have been published by Nature, while Radiology and The Lancet Digital Health each have published 3.

**Table 5:** Top 20 most cited documents on ChatGPT.

**No**.	**Title**	**Total citation**	**Article type**	**Journal**	**Country of first author**	**References**
1	ChatGPT is fun, but not an author	93	Editorial	Nature	USA	Thorp, 2023
2	ChatGPT listed as author on research papers: many scientists disapprove	88	Note	Nature	UK	Stokel-Walker, 2023
3	ChatGPT: five priorities for research	63	Note	Nature	Netherlands	van Dis et al., 2023
4	Tools such as ChatGPT threaten transparent science; here are our ground rules for their use	61	Editorial	Nature	USA	Editorial, 2023
5	Abstracts written by ChatGPT fool scientists	57	Note	Nature	USA	Else, 2023
6	ChatGPT and Other Large Language Models Are Double-edged Swords	47	Editorial	Radiology	USA	Shen et al., 2023
7	AI bot ChatGPT writes smart essays—should professors worry?	43	Note	Nature	UK	Stokel-Walker, 2022
8	ChatGPT and the Future of Medical Writing	33	Note	Radiology	USA	Biswas, 2023
9	What ChatGPT and generative AI mean for science	33	Note	Nature	UK	Stokel-Walker and Van Noorden, 2023
10	How Does ChatGPT Perform on the United States Medical Licensing Examination? The Implications of Large Language Models for Medical Education and Knowledge Assessment	33	Article	JMIR Medical Education	USA	Gilson et al., 2023
11	Generating scholarly content with ChatGPT: ethical challenges for medical publishing	29	Note	The Lancet Digital Health	Switzerland	Liebrenz et al., 2023
12	ChatGPT: the future of discharge summaries?	28	Note	The Lancet Digital Health	UK	Patel and Lam, 2023
13	Collaborating With ChatGPT: considering the Implications of Generative Artificial Intelligence for Journalism and Media Education	28	Article	Journalism and Mass Communication Educator	USA	Pavlik, 2023
14	“So what if ChatGPT wrote it?” Multidisciplinary perspectives on opportunities, challenges and implications of generative conversational AI for research, practice and policy	24	Article	International Journal of Information Management	India	Dwivedi et al., 2023a
15	Can artificial intelligence help for scientific writing?	21	Article	Critical Care	Belgium	Salvagno et al., 2023
16	ChatGPT: evolution or revolution?	19	Editorial	Medicine, Health Care, and Philosophy	Ireland	Gordijn and Have, 2023
17	ChatGPT: friend or foe?	18	Editorial	The Lancet Digital Health		The Lancet Digital Health, 2023
18	Chatting about ChatGPT: how may AI and GPT impact academia and libraries?	16	Review	Library Hi Tech News	USA	Lund and Wang, 2023
19	ChatGPT Is Shaping the Future of Medical Writing But Still Requires Human Judgment	15	Editorial	Radiology	Brazil	Kitamura, 2023
20	What Does ChatGPT Say: The DAO from Algorithmic Intelligence to Linguistic Intelligence	15	Review	IEEE/CAA Journal of Automatica Sinica	China	Wang et al., 2023

The citation analysis of authors visualizes the most cited authors and their citation networks. It is observed that 187 authors have at least 10 citations. Stokel-Walker, C. is the most cited author, followed by Thorp, H. H., Bockting, C. L., and Else, H. with 93, 63, and 57 citations, in respective order (Figure 4B). Besides having the most citations, Stokel-Walker, C. has the largest citation network with 93 citing partners. The second largest citation network is associated with Biswas, S. with 88 different citing partners. The most frequent citing partners are Wu, H. and Cheng, K., who cited each other at least 13 times, the next in line are Wu, H. and Lu, Y., having 12 link strength.

The citation analysis of countries showed that a large number of countries are actively citing each other's work. There are 38 countries that have received at least 10 citations, 29 countries that have received at least 20 citations, and 10 countries that have received at least 50 citations. The citation network of countries is very dense, meaning that there are a lot of connections between countries (Figure 4C). The most citing partners are the United States and the United Kingdom, with a link strength of 31. The United States and India are the second most citing partners, with a link strength of 30. The United States and Australia are the third most citing partners, with a link strength of 25. In terms of citation network, the United States has the largest network, with 35 links. India and China are tied for second place, with 30 links each.

The citation network analysis of journals revealed that the largest citation network consisted of 22 journals citing each other frequently (Figure 4D). Twenty-first journals have at least 10 citations. The most cited journal on the topic of ChatGPT is Nature, with 367 citations, followed by Radiology, with 100 citations, and Science, with 93 citations. Nature and Annals of Biomedical Engineering are the most frequent citing partners (Link strength 6). Afterward, Nature, along with Healthcare, Radiology, and Library Hitech News, makes the next frequent citing partners citing each other at least four times.

### 3.3 Collaboration network of author, institution, and countries

**Figure 5:**
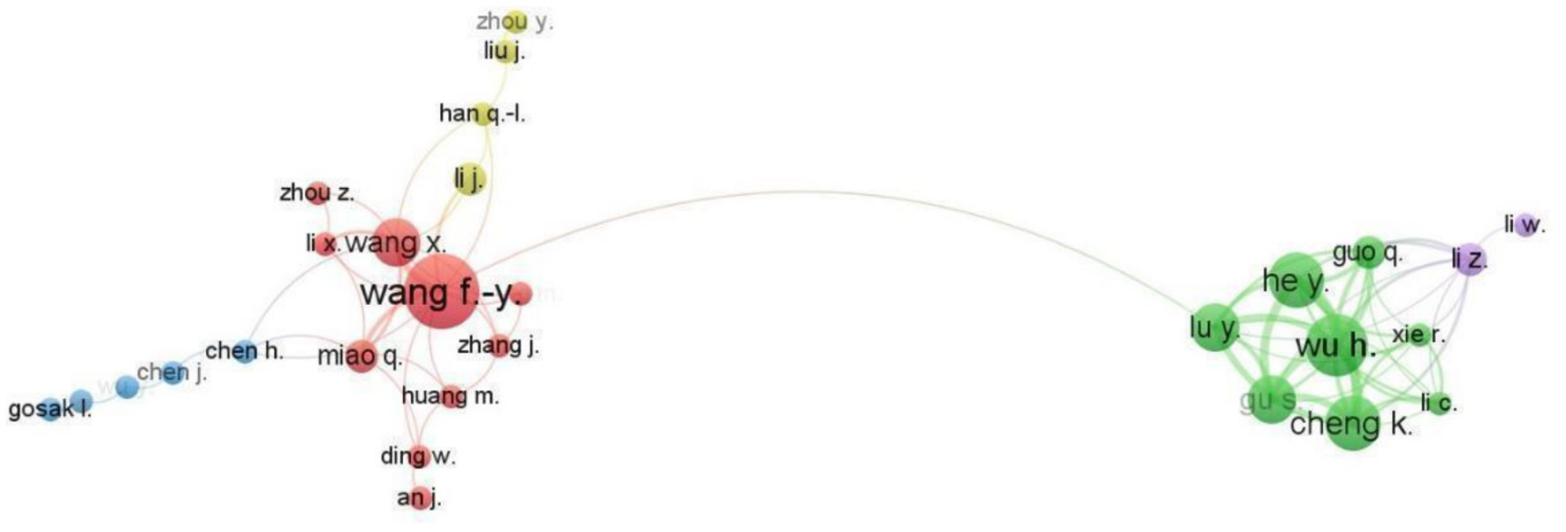
Largest collaboration network of authors.

Of the 118 authors who have published at least two articles on ChatGPT, only 29 have collaborated with each other. These 29 authors are divided into five clusters, with the largest cluster (cluster 1) consisting of 10 authors. The second largest cluster (cluster 2) consists of eight authors, followed by cluster 3 (five authors), cluster 4 (four authors), and cluster 5 (two authors; Figure 5). The two most collaborative authors, Wang, F. Y. and Wang, X, belong to cluster 1 with 11 (17 link strengths) and 9 (14 link strengths) collaborations, respectively. Afterward, Li, Z., with a link strength of 13 and 9 collaboration, contributed to the 5th cluster. All eight authors of green cluster Wu, H., Quo, Q., Hey, Y., Lu, Y., Gu, S, Cheng, K, Li, C and Xie, R., have eight collaborations each. Wu, H with Cheng, K., and Hey, Y. are the most frequent collaborating partners (Link strength). Wu, H. and Gu, S. are the second most collaborative partners with a link strength of 5.

**Figure 6:**
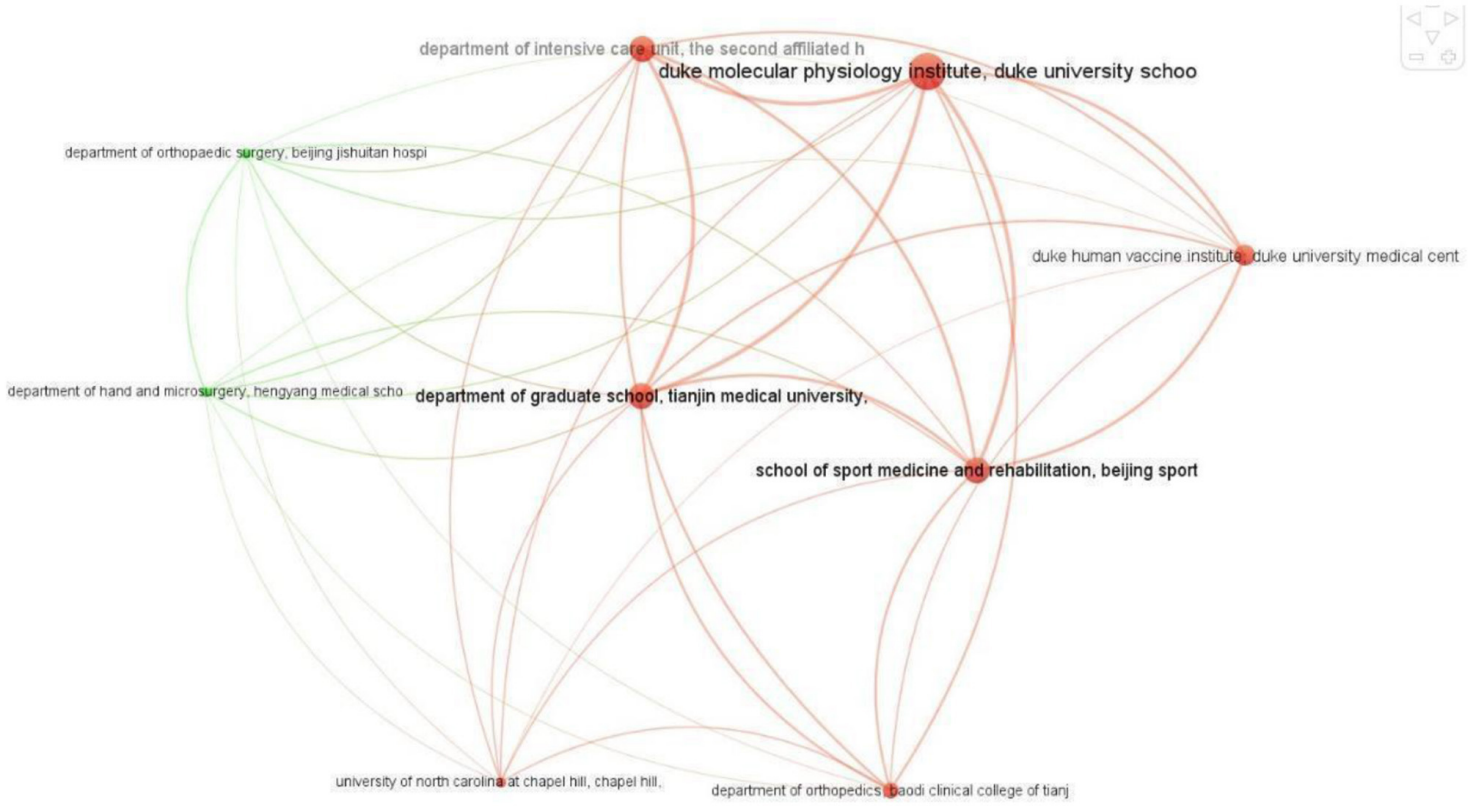
Largest collaboration network of institutions.

Out of 1,195 institutions around the world, 53 have published at least two articles on ChatGPT. The largest collaborating network consists of only nine institutions, six from China and three from the United States (Figure 6). All nine institutions have an equal number of collaborating links, with eight each. However, *Duke Molecular Physiology Institute, Duke University School of Medicine, Durham, NC, United States*, has published the most articles on ChatGPT in collaboration with eight different institutions. It is followed by the *Department of Graduate School, Tianjin Medical University, Tianjin, China, the Department of Intensive Care Unit, The Second Affiliated Hospital of Zhengzhou University, Henan, Zhengzhou, China*, and the *School of Sport Medicine and Rehabilitation, Beijing Sport University, Beijing, China*, which have published five articles each.

**Figure 7:**
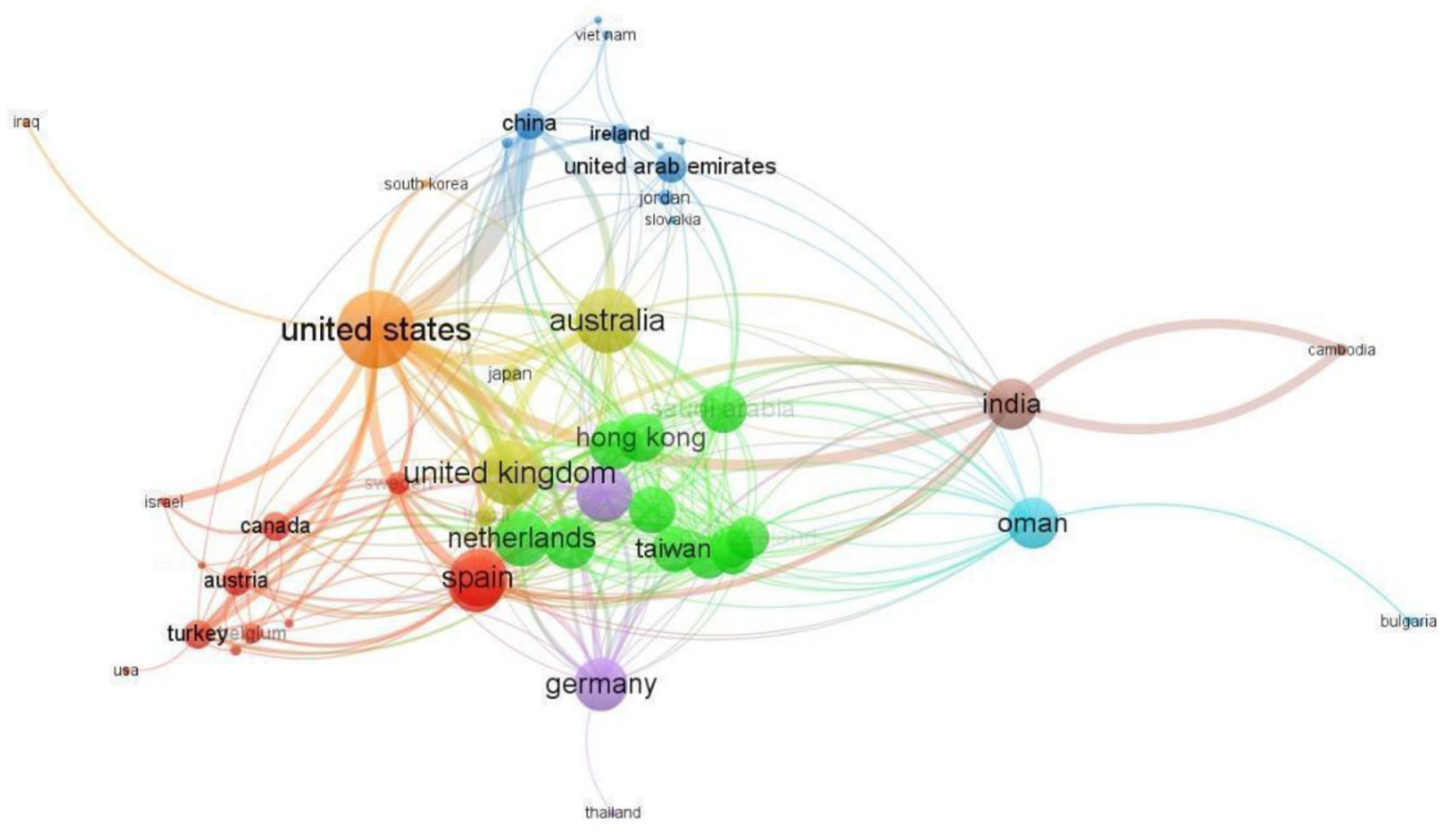
Largest collaboration network of countries.

**Figure 8:**
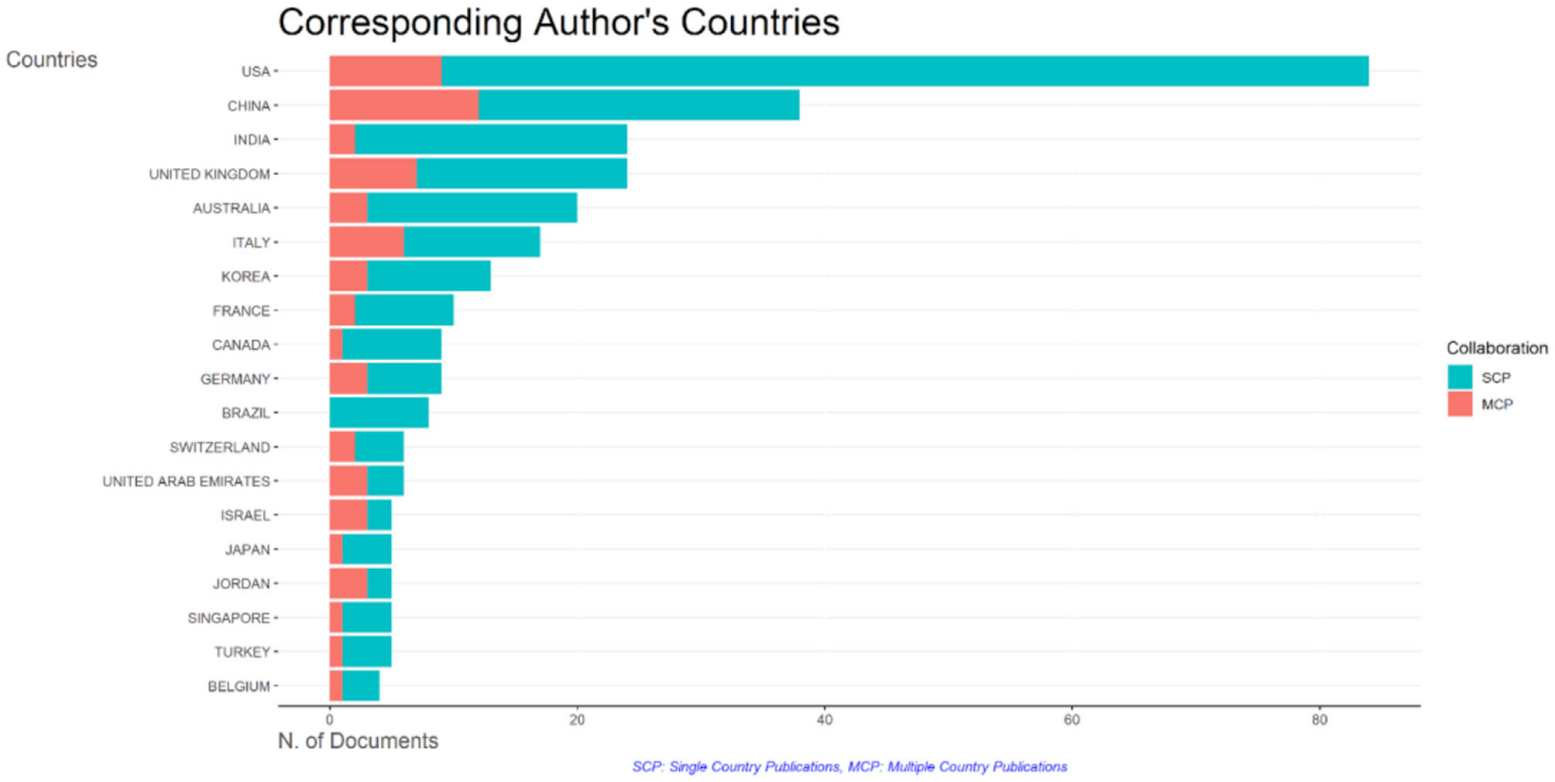
Graph representing single country and multiple country publications on ChatGPT.

A total of 56 countries have published at least two papers on ChatGPT, and 53 countries have collaborated with each other on these papers. The United States has the most collaborating partners (35 countries), with China being the most frequent collaborator (link strength of 12; Figure 7). The United Kingdom, India, Australia, and Italy are also frequent collaborators (link strength of 7 each). Australia and the United Kingdom have the second most collaborating partners (29 countries each). Afterward, India and Nigeria, and India and Cambodia are the most frequent collaborators (link strength of 6 each). The United States also leads in single-country publications (SCP) with 75 articles, followed by China and India with 25 and 22 articles, respectively (Figure 8). China has the highest number of multiple-country publications (MCP) with 12 articles, followed by the United States with nine articles. The United Kingdom and Italy have 7 and 6 MCPs, respectively.

We found a lot of inter-disciplinary and intra-disciplinary collaboration in ChatGPT research. The data also indicate that there is much institutional collaboration exploration. The institutions with the most collaboration tend to be located in countries with strong ties with other countries in research and education, such as Australia, Canada, and the United States. These institutions also have a diverse and global faculty and student body, which facilitates cross-cultural and cross-national exchanges.

### 3.4 Sankey diagram (three-field plot)

**Figure 9:**
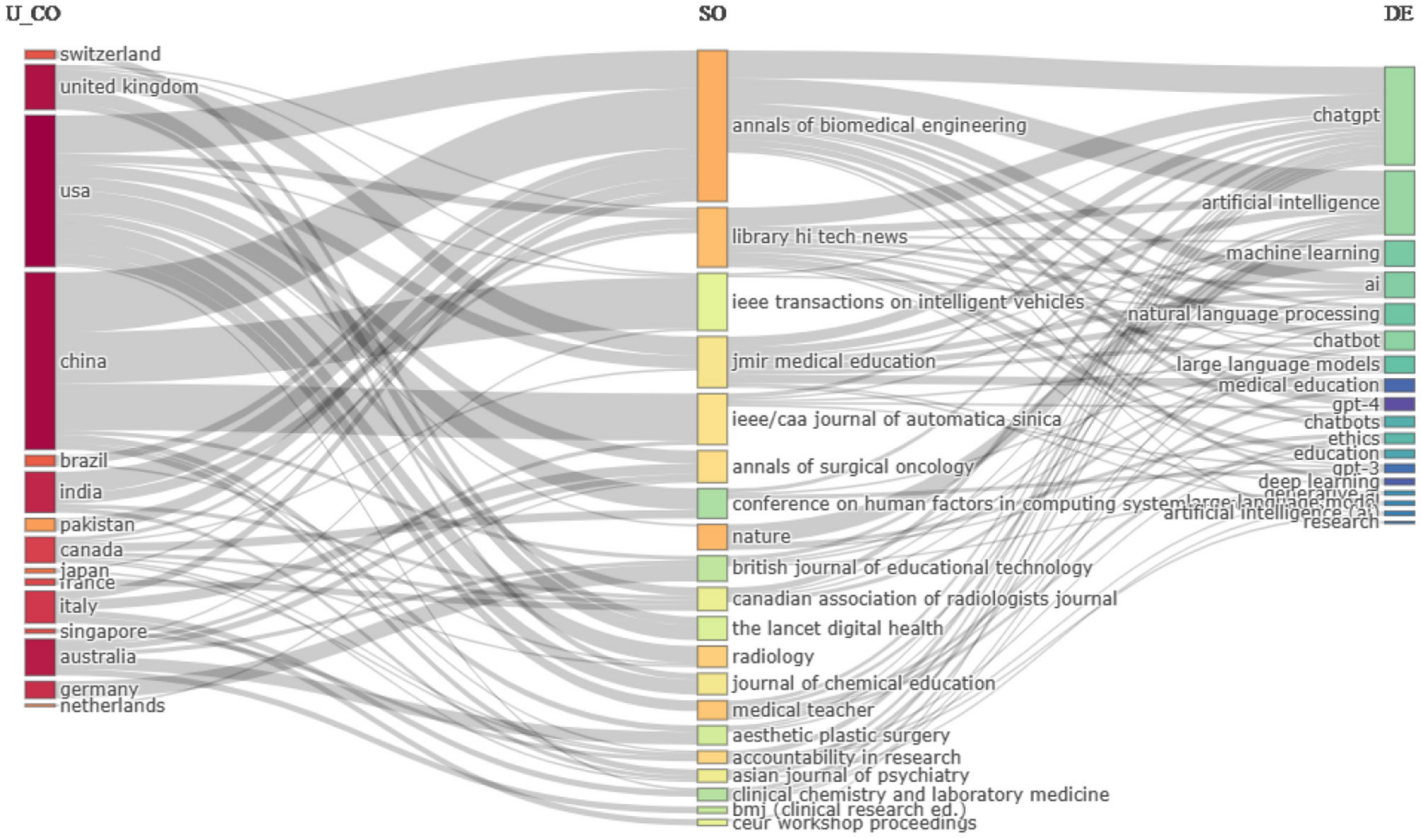
Three-field plot of countries, journals, and author's keywords. AU_CO, Author's countries; SO, source; and DE, Author's keywords.

A Sankey diagram, also known as a three-field plot, is used to visualize the flow of values from one set to another. These plots are used to depict the relationships and data transitions between three distinct categories, providing a three-dimensional perspective on the evolution and exchange of information among these categories. These graphical representations showcase data from three distinct sources, using lines to represent the links between the fields. The width of these lines illustrates the quantity or strength of the connections. Figure 9 illustrates the relationship among the author's country, sources of their publications, and keywords chosen by them. The analysis reveals that authors from the United States have published their articles in 12 different journals, indicating a wide range of publication sources compared to other countries. In contrast, Chinese authors predominantly publish their work in three journals: Annals of Biomedical Engineering, IEEE Transactions on Vehicles, and IEEE/CAA Journal of Automatica Sinica. Canadian and Australian authors exhibit the next highest levels of publication diversity, with seven and six different journals, respectively.

Annals of Biomedical Engineering is a favored choice among authors from seven different countries, with the majority of publications coming from China, the United States, and India, in respective order. The most commonly selected keywords by authors include “ChatGPT,” “artificial intelligence,” “natural language processing,” “large language model,” “chatbot,” and “machine learning.” Notably, the most diverse keyword is “ChatGPT,” followed by “artificial intelligence,” which is highly popular among authors as well as sources. Among the journals, Library Hi Tech News has indexed 13 out of the top 20 most frequently used author's keywords, whereas Nature has only three keywords in common with the author's keywords, viz., “machine learning,” “ethics,” and “education.”

**Figure 10:**
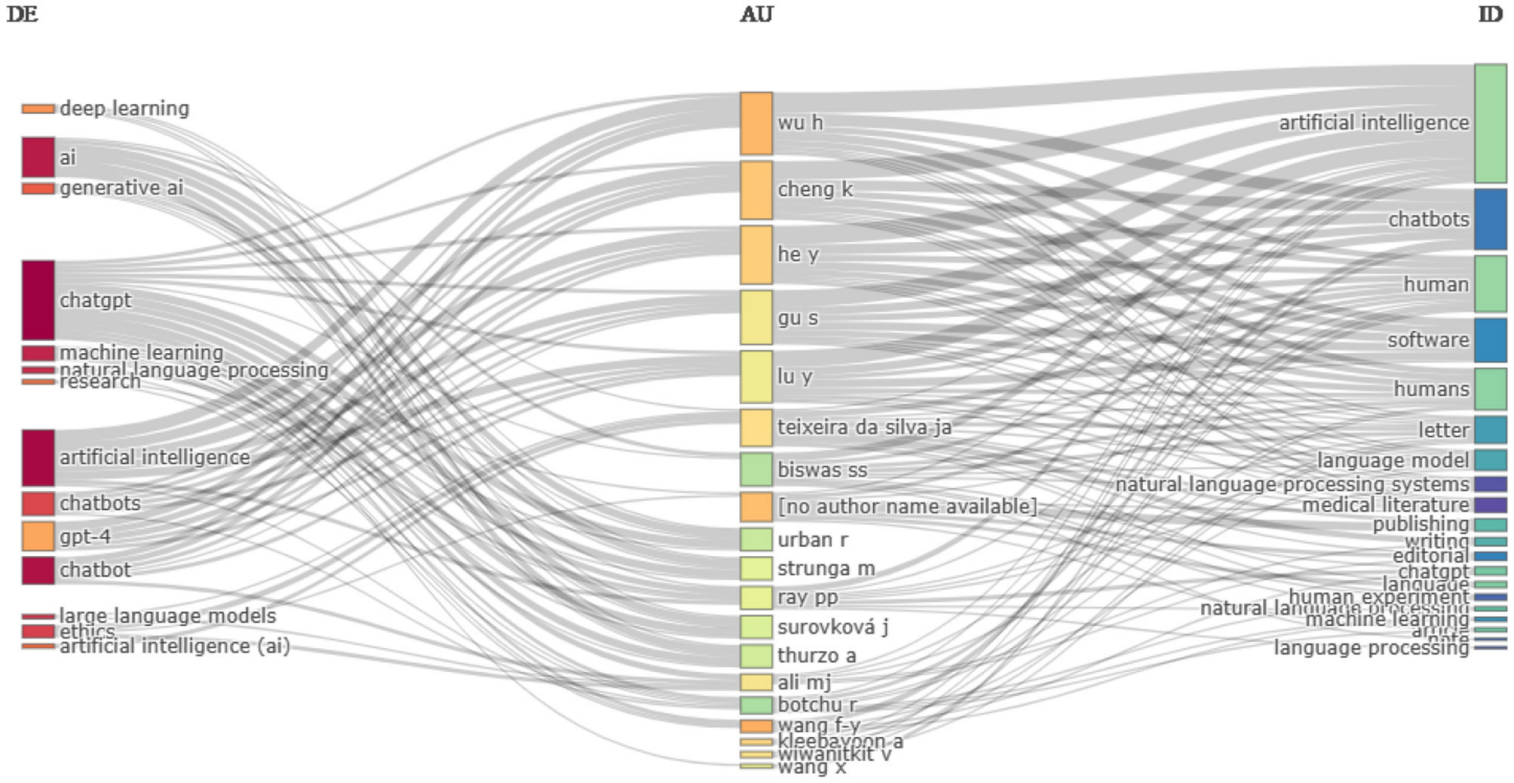
Three-field plot of Keyword Plus, authors and author's keywords. DE, Author's keywords; AU, Author; and ID, Keyword Plus.

Figure 10 shows the relationship between author keywords, authors, and Keywords Plus. Author keywords are chosen by authors, while Keywords Plus is automatically chosen by journals based on the frequency of cited and referenced title words. It is observed that author keywords and Keywords Plus are quite different from each other. For example, “ChatGPT” is the most frequently used keyword by authors, while Keywords Plus tends to favor “artificial intelligence.” There are some common keywords in both categories, but their frequencies vary. For example, “ChatGPT” is a favorite choice of authors, but it is one of the least appearing according to Keywords Plus. Notably, authors such as Wu, H., Cheng, K., Hey, Y., Gu, S., and Lu, Y. share common keywords that fall under both keyword categories, viz., “artificial intelligence,” “chatbot,” and “chatbots.”

**Figure 11:**
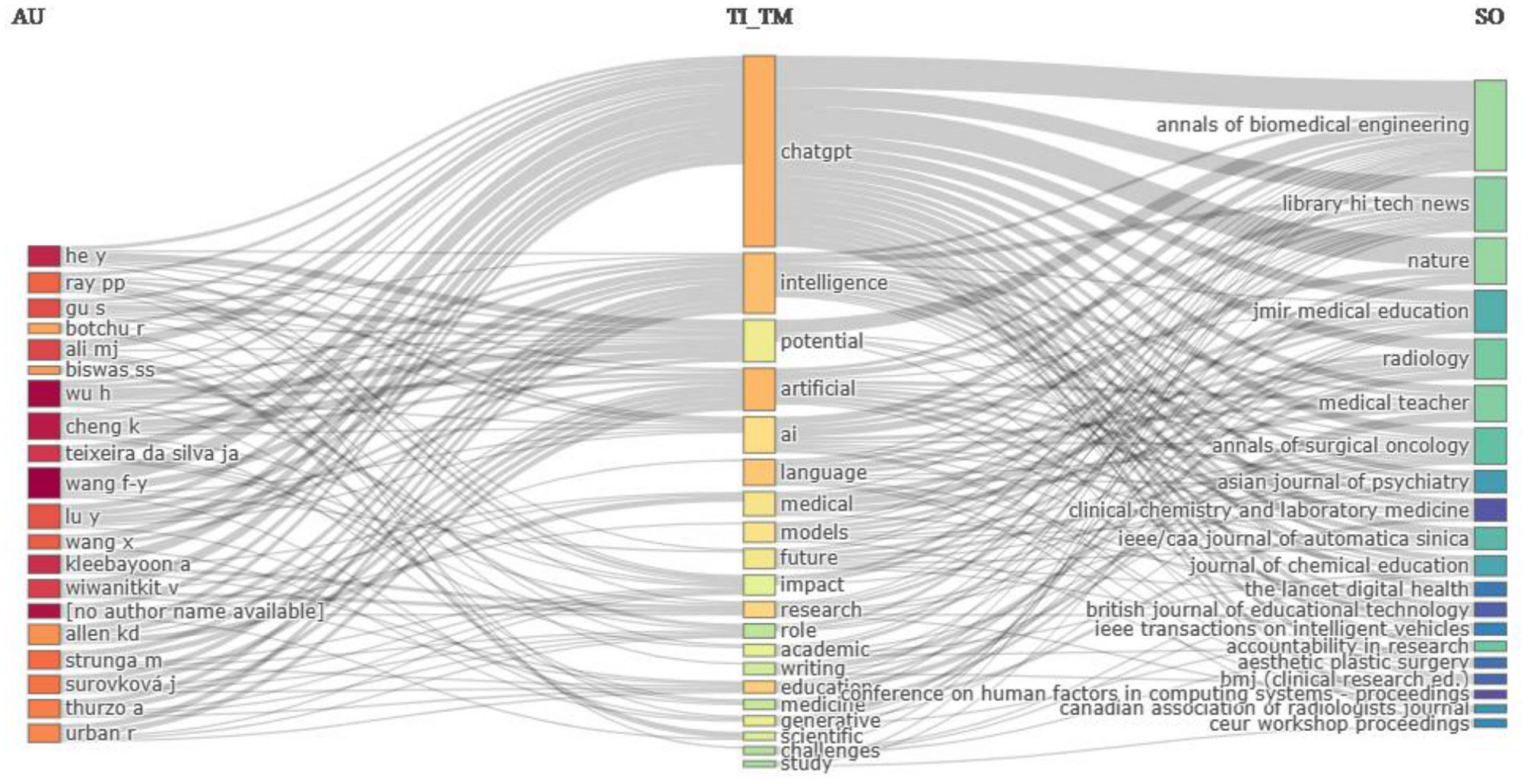
The three-field plot of authors, title-terms, and sources. AU, Author; Tl_TM, Title-term; and SO, Source.

The three-field plot Sankey diagram (Figure 11) shows the relationship between the author, title-term used by them, and sources. It is obvious that “ChatGPT” is the most widely used title term by the authors as well as the most widely accepted title term of journal publications. Terms like “Intelligence” and “potential” in the titles of publications show the trending research topics related to the ChatGPT. Apart from machine learning-related terms such as “AI,” “language,” “model,” “artificial,” and “intelligence,” the most frequent title terms are “medical,” “academic,” “writing,” “education,” “medicine” etc. reflecting the recent thrust area of ChatGPT research. These title-terms are very frequently accepted by top journals like Nature, Annals of Biomedical Engineering, Radiology, and Library Hi Tech News.

### 3.5 Keyword analysis

**Figure 12:**
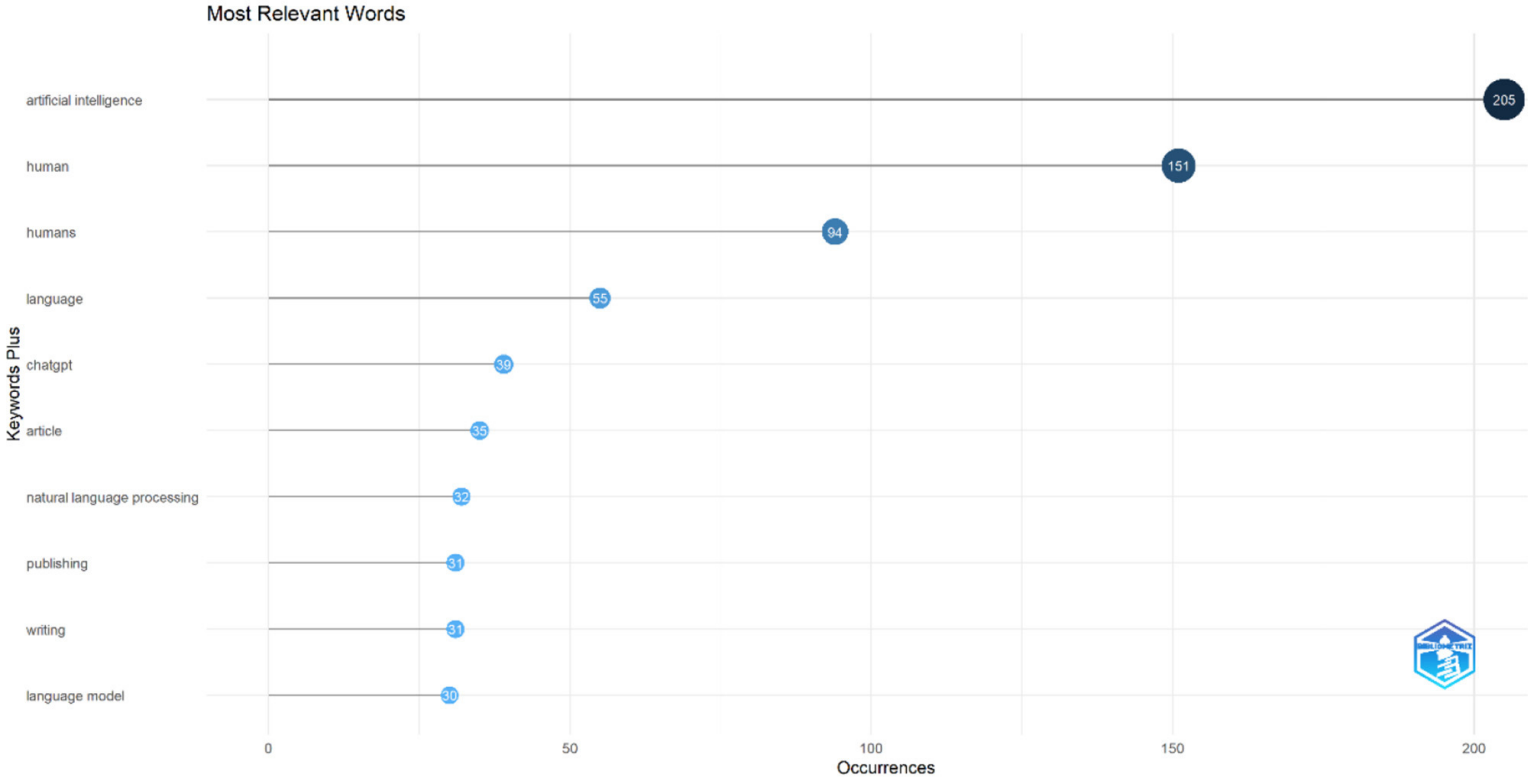
Most relevant keywords and their occurrences.

**Figure 13:**
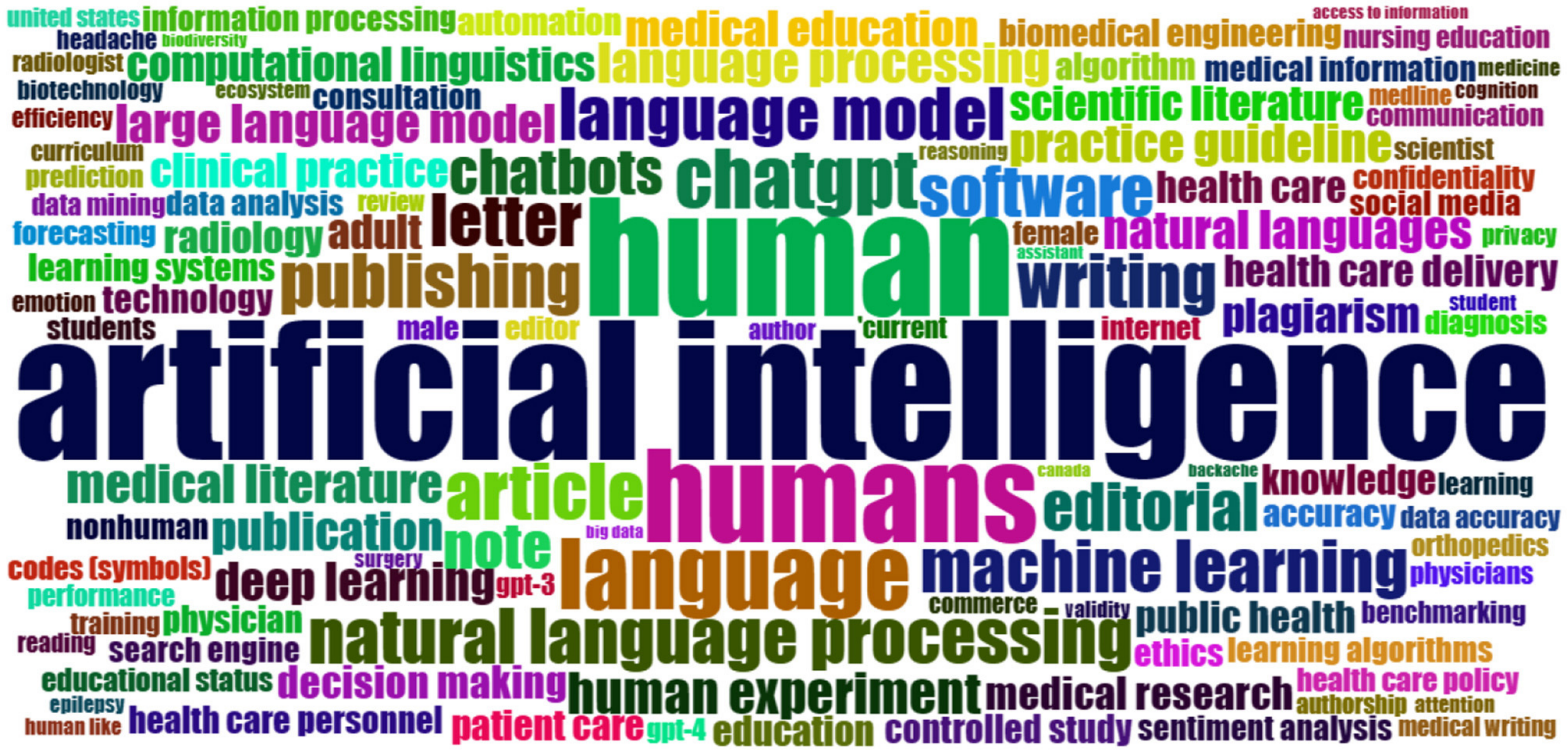
Word cloud of keywords on ChatGPT.

For keyword analysis, the most relevant keywords are retrieved using Bibliometric software package. As Figure 12 shows, the most occurring keyword is “artificial intelligence” with 205 occurrences, followed by “human,” “humans,” “language,” and “chatgpt” with 151, 94, 55, and 39 occurrences, respectively. Other keywords with high occurrences are “article,” “natural language processing,” “publishing,” and “writing.” Additionally, a word cloud of the most frequent keywords is plotted to illustrate the highly used terms in the field of ChatGPT research (Figure 13).

**Figure 14:**
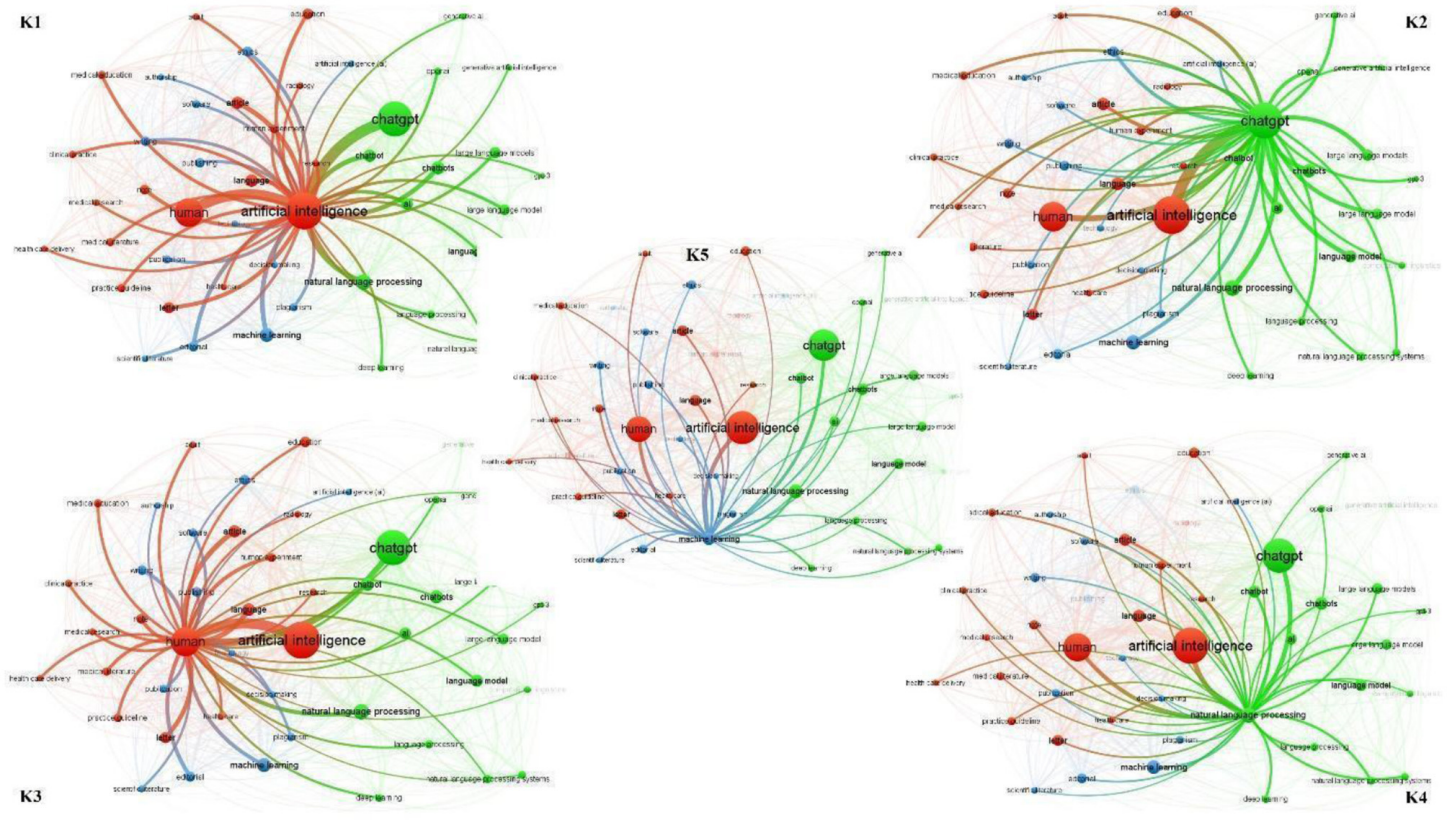
Top 5 most occurred keywords and co-occurred keyword their network.

To conduct co-occurrence analysis of keywords, a threshold of at least 10 occurrences was chosen, resulting in a selection of 49 keywords that appeared at least 10 times. Synonyms of the keywords were excluded, and network maps were generated to visualize the top five most frequently occurring keywords and their co-occurring keywords. It was observed that “artificial intelligence” (Figure 14 K1) and “chatgpt” (Figure 14 K2) were the two most commonly co-occurring keywords in ChatGPT literature, each appearing alongside 47 distinct keywords. The third and fourth most frequently co-occurring keywords were “human” (Figure 14 K3) and “natural language processing” (Figure 14 K4), with co-occurrence network strengths of 46 and 43, respectively. “Machine learning” secured the fifth position, co-occurring with 39 different keywords in the network (Figure 14 K5).

Furthermore, Table 6 presents the top 10 pairs of keywords with the highest frequency of co-occurrence. The pair “artificial intelligence” and “chatgpt” exhibited the most frequent co-occurrence, appearing together 117 times. The second most frequent pair was “artificial intelligence” and “human,” which co-occurred 102 times. Additionally, “human” and “chatgpt” were found to co-occur 38 times.

**Table 6:** Top 10 pairs of co-occurred keywords.

**No**.	**Keyword 1**	**Keyword 2**	**Co-occurrence**
1	Artificial intelligence	ChatGPT	117
2	Artificial intelligence	Human	102
3	Human	ChatGPT	38
4	Artificial intelligence	Natural language processing	33
5	Human	Article	32
6	Artificial intelligence	Machine learning	29
7	Artificial intelligence	Article	28
8	ChatGPT	Natural language processing	27
9	Artificial Intelligence	Chatbot	27
10	ChatGPT	Chatbot	27

## 4 Discussion

### 4.1 Outbreak

The comprehensive analysis of ChatGPT research conducted in the period from November 2022 to early June 2023 reveals a thriving research interest in the field. During this short timeframe, a total of 533 documents were produced, indicating a significant surge in scholarly publications related to ChatGPT. The rapid surge in the number of publications highlights the growing interest in the potential applications of ChatGPT and the profound influence it has had on humans across the globe. Google Trends for the search term “ChatGPT” during this same period shows how the interest in the topic increased gradually over time. The level of interest reached a peak in April 2023, and the interest remained relatively stable until the end of May 2023 (see Supplementary material).

### 4.2 Collaboration patterns

Collaboration among researchers is evident in the analysis, with a high collaboration rate of 88.91% observed among the authors. This suggests a strong community of researchers working on ChatGPT who are actively sharing ideas and resources to advance the field. The involvement of 1,195 institutions from various countries highlights research collaboration. This reflects the ability of technological innovations and artificial intelligence to unite researchers from diverse backgrounds for interdisciplinary work. Additionally, it underscores the motivation to be a “first mover” in new scientific fields, with collaboration facilitating rapid and impactful publication. This can be seen in light of the findings of Sabatier and Chollet (2017), who found that pioneers in new research fields have, over time, had higher scientific production.

### 4.3 Document types

The type of documents published on ChatGPT shows a diverse range of contributions. Empirical papers constitute the largest portion of the documents, followed by letters, editorials, and notes. The significant presence of letters, notes, and editorials within the corpus indicates that there is a variety of perspectives and opinions surrounding ChatGPT. This also underscores the level of interest and excitement surrounding the rise of ChatGPT. Furthermore, these types of outputs are also a route to swift publication, which benefits authors not only in terms of enabling themselves to gain recognition as key thinkers within the field but also benefit from a potential surge in citations. However, it should be noted that researchers should exercise caution when citing and referring to work that is published in outlets that are not peer-reviewed, as the information contained could be misleading in some cases, leading to a flawed impression of this emerging field. We also uncovered that the number of review articles published is relatively low, suggesting an area for further exploration and synthesis of existing knowledge.

### 4.4 Journals

The analysis of the top journals reveals the leading platforms for ChatGPT research. Annals of Biomedical Engineering published the highest number of articles, followed by Nature and Library Hi Tech News. Nature also stands out as the most cited journal, indicating its influence and reputation in the field. Given the wide-ranging implications of ChatGPT we would expect the list of journals that feature relevant research to expand exponentially over the coming months and years as the understanding of the implications of this innovation improves over time. In terms of countries, the United States emerges as the most prolific contributor with the highest number of publications. India and the United Kingdom follow closely behind. The USA also demonstrates the highest citation count, indicating its global academic impact. Other countries such as Australia, China, and Italy have also made significant contributions to ChatGPT research.

### 4.5 Authors

The top authors in the field showcase their contributions and impact. Wang F. Y. from the Institute of Automation Chinese Academy of Sciences leads with the highest article count, while authors from Duke University School of Medicine in the USA also feature prominently. Notably, authors with a lower article count have achieved significant citation numbers, highlighting the quality and impact of their work. The top institutions contributing to ChatGPT research represent a mix of organizations from different countries. Duke University participates in the highest number of papers, followed by the Chinese Academy of Sciences and Chandigarh University. Duke University also received the highest number of citations, indicating the institution's research excellence and impact. The analysis of citation networks reveals the most cited documents, authors, countries, and journals. “ChatGPT is fun, but not an author” by Thorp (2023) emerges as the most cited document, followed by “ChatGPT listed as author on research papers: many scientists disapprove” by Stokel-Walker (2023). These documents highlight the discussions and controversies surrounding ChatGPT and its use in research. The presence of notes and editorials in the top cited documents suggests that discussions and opinions are driving the conversation in the field.

### 4.6 Keywords

Keyword analysis is an essential aspect of bibliometric research, providing insights into the most relevant terms and their co-occurrence patterns (Farhat et al., 2023c). In the case of ChatGPT, a bibliometric software package was utilized to retrieve the most occurred keywords. Among these keywords, “Artificial intelligence” emerged as the most frequent, appearing 205 times. Following closely were “human,” “humans,” “language,” and “ChatGPT” with 151, 94, 55, and 39 occurrences, respectively. The emergence of “human” and “humans” as significant co-occurring keywords is important given the nature of the innovation. In a world where artificial intelligence is taking over and automating many processes, there is considerable concern about its impact on human nature, which could result in significant political and economic issues if not addressed and considered carefully. For example, even before the emergence of ChatGPT, Hassani et al. (2020) argued the importance of focusing on intelligence augmentation as the way forward and the urgent need for ethical frameworks that can regulate the growth of AI whilst protecting the wellbeing and interest of humans. Other significant keywords included “article,” “natural language processing,” “publishing,” and “writing.”

## 5 Conclusion

### 5.1 Implications

In this paper, we have carried out a comprehensive bibliometric analysis of the scholarly footprint of ChatGPT, zooming in on the early outbreak phase. Our findings have theoretical, methodological, practical, societal, and ethical implications.

From a theoretical perspective, our study provides an interesting view of the early developments in the establishment of a research field around a new technology. By employing bibliometric and scientometric methods, we have explored various dimensions of ChatGPT research, including overall publication trends, citation patterns, collaborative networks, application domains, and possible future directions.

The analysis of publication trends revealed a remarkable surge in scholarly output related to ChatGPT within a short time frame of about 6 months. The analysis also examines the publication venues contributing to ChatGPT research and evidences the impact of ChatGPT on diverse scientific disciplines. Furthermore, the study explores the contributions of different countries to ChatGPT research and finds that the United States has the most significant global academic impact in the field of ChatGPT, but other countries such as China, Australia, and Italy have also made notable contributions to ChatGPT research. In terms of influential authors, Wang F. Y. from the Chinese Academy of Sciences and Wu H. from Duke University are among the top authors based on article count and total citations.

In terms of practical implications, our study serves as a valuable resource for researchers and other experts involved in the broader AI field, offering a comprehensive understanding of the scholarly footprint of ChatGPT. It can serve as a quick reference guide for new researchers who orient themselves in the landscape of GPT research by highlighting the most influential authors, studies, and institutions thus far. Moreover, the findings can guide future research endeavors, collaborations, and innovations in enhancing ChatGPT's capabilities and impact. By mapping early research on ChatGPT and identifying trends, we aim to stimulate discussions and contribute to the continuous advancement of ChatGPT and its applications across domains.

Finally, our study has societal and ethical implications. The bibliometric analysis clearly demonstrates that ChatGPT, in just a span of a few months, garnered much attention among researchers working across different scholarly fields (Sohail, 2023). Many commentators have pointed out societal and ethical issues related to ChatGPT and similar AI models (Rahimi and Abadi, 2023; Zhuo et al., 2023; see, for example, Farina and Lavazza, 2023), such as bias and fairness, privacy concerns, employment impact, over-reliance on technology, and security risks. Some have even gone as far to suggest that AI can be an existential threat to humanity (Chomsky et al., 2023; Harari, 2023). From a bibliometric perspective, it becomes interesting to follow the extent to which societal and ethical aspects will become key areas of future ChatGPT research or whether researchers will primarily focus on technical and instrumental aspects.

### 5.2 Limitations and directions for future research

Like any study, our analysis has certain limitations that should be considered carefully. First, our study relies on bibliometric and scientometric methods that are mostly quantitative and provide limited qualitative insight. Therefore, follow-up studies could employ content analysis of the most influential articles or gather primary data via interviews with authors and experts involved in the AI field, which may enable a more multifaceted analysis. Second, bibliometric analyses might also be subject to other biases, such as self-citations. Future studies could examine publication patterns in more detail, in particular the role played by prolific researchers and journals that specialize in publishing research on “hot” topics such as ChatGPT to boost their own citation metrics and impact factors. It would also be interesting to follow the scientific trajectories of some of the “first movers” in the field of ChatGPT research to see if they gain long-term career advantages and increased scientific productivity (Sabatier and Chollet, 2017). The same can possibly be done at the journal level to explore whether journals with high market shares of ChatGPT publications obtain increased citation metrics or other reputational effects in the long run.

Another limitation of our bibliometric analysis is that based on bibliometric data collected and analyzed at one point in time. Therefore, it is difficult to make predictions about the future evolution of the research literature on ChatGPT. Donthu et al. (2021, p. 295) point out that “bibliometric studies can only offer a short-term forecast of the research field” and highlight that it is important to be careful when making assertions about its future importance and impact. This is especially the case regarding research on ChatGPT since it has been rapidly expanding, and there is much uncertainty related to its future evolution and trajectory. Therefore, it becomes of great importance to revisit and update the findings of this bibliometric study. Such updated analyses can also shed light on the dynamics and evolution of the scientific field and community involved in the field of ChatGPT research.

## Data availability statement

Publicly available datasets were analyzed in this study. This data can be found at: https://www.scopus.com/.

## Author contributions

FF: Conceptualization, Data curation, Formal analysis, Investigation, Resources, Software, Visualization, Writing – original draft, Writing – review & editing. ES: Resources, Validation, Visualization, Writing – original draft, Writing – review & editing. HH: Resources, Validation, Writing – original draft, Writing – review & editing. DM: Project administration, Resources, Validation, Writing – original draft, Writing – review & editing. SS: Conceptualization, Project administration, Resources, Writing – original draft, Writing – review & editing. YH: Resources, Validation, Writing – original draft, Writing – review & editing. MA: Resources, Validation, Writing – review & editing. AZ: Resources, Validation, Writing – review & editing.
