# Supplementary material for: The scholarly footprint of ChatGPT: a bibliometric analysis of the early outbreak phase
Source: Front Artif Intell. 2024 Jan 5;6:1270749. doi: 10.3389/frai.2023.1270749 (PMC10797012; doi:10.3389/frai.2023.1270749)
Supplement: Supplementary file 1 [file Table_1.docx]

**Table: Overview of the retrieved data related to ChatGPT.**

| **Description** | **Results** |
| --- | --- |
| **Timespan** | 2022-2023 (November 2022 to Early June 2023) |
| **Sources (Journals, Books, etc.)** | 341 |
| **Documents** | 533 |
| **Annual Growth Rate %** | 17566.67 |
| **Total citations** | 1362 |
| **Self-Citations** | 824 |
| **Self-Citations %** | 60.5 |
| **Average citations per doc** | 2.546 |
| **References** | 11244 |
| **DOCUMENT CONTENTS** |  |
| **Total Keywords** | 1998 |
| **Keywords Plus (ID)** | 1371 |
| **Author's Keywords (DE)** | 882 |
| **AUTHORS** |  |
| **Authors** | 1434 |
| **Authors of single-authored docs** | 159 |
| **Single-authored docs** | 182 |
| **Single-authored docs %** | 34.14 |
| **Co-Authors per Doc** | 3.08 |
| **Authors collaboration %** | 88.91 |
| **COUNTRIES** |  |
| **Countries** | 87 |
| **Single-country docs** | 420 |
| **Multiple-country docs** | 113 |
| **Countries collaboration %** | 21.2 |
| **INSTITUTIONS** |  |
| **Institutions** | 1195 |
| **Institutions collaboration %** | 6.44 |
