# Supplementary figures and images for: The scholarly footprint of ChatGPT: a bibliometric analysis of the early outbreak phase
Source: Front Artif Intell. 2024 Jan 5;6:1270749. doi: 10.3389/frai.2023.1270749 (PMC10797012; doi:10.3389/frai.2023.1270749)

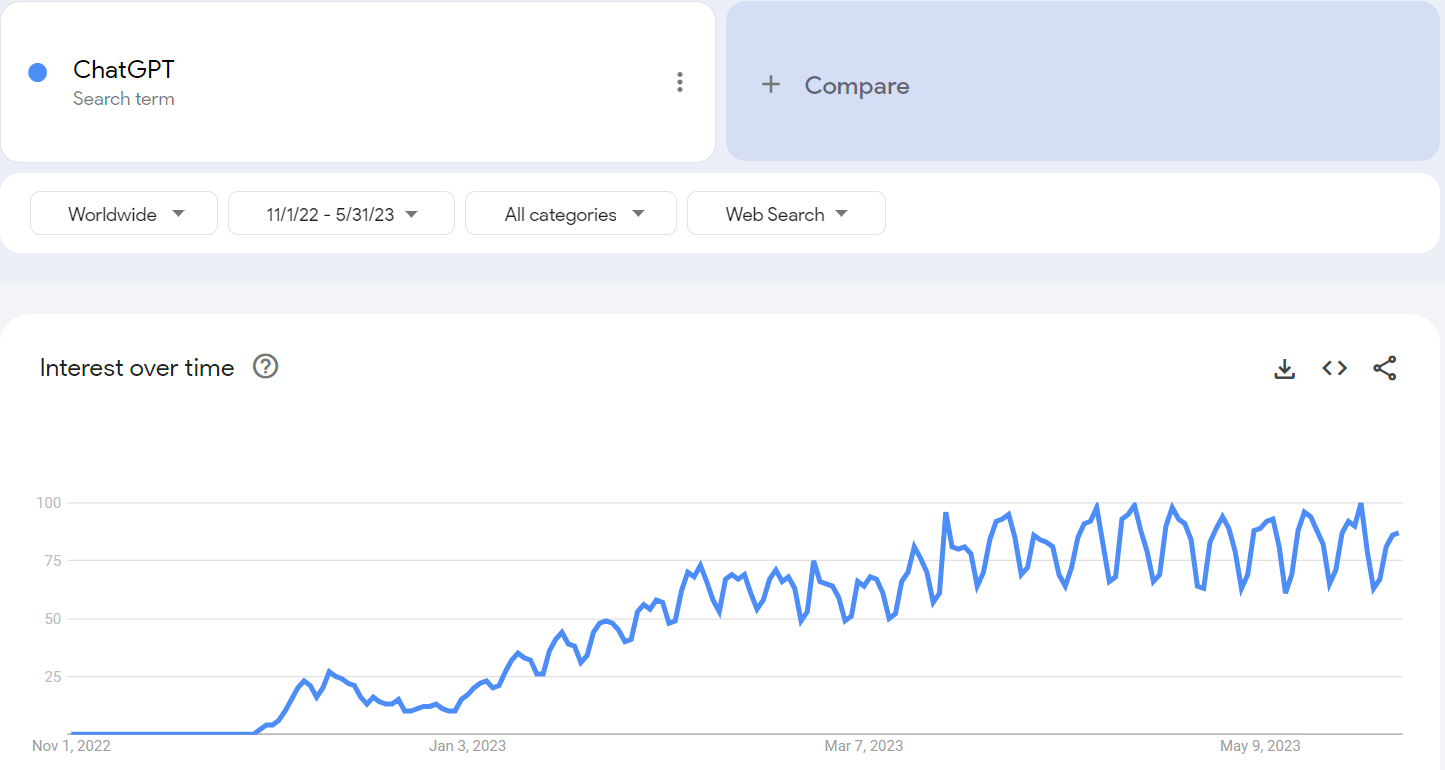


**Figure: Worldwide Google Trends for “ChatGPT” from 1st November 2022 - 31st May 2023.**

Supplement: Supplementary file 2 [file Data_Sheet_1.docx]
